# Automated detection of cardiac rest period for trigger delay calculation for image-based navigator coronary magnetic resonance angiography

**DOI:** 10.1186/s12968-023-00962-9

**Published:** 2023-10-02

**Authors:** Gregory Wood, Alexandra Uglebjerg Pedersen, Karl P. Kunze, Radhouene Neji, Reza Hajhosseiny, Jens Wetzl, Seung Su Yoon, Michaela Schmidt, Bjarne Linde Nørgaard, Claudia Prieto, René M. Botnar, Won Yong Kim

**Affiliations:** 1 Department of Cardiology, Aarhus University Hospital, Palle Juul Jensens Boulevard 99, 8200 Aarhus N, Denmark; 2 Department of Clinical Medicine, Aarhus University, Aarhus, Denmark; 3 MR Research Collaborations, Siemens Healthcare Limited, Camberley, UK; 4 School of Biomedical Engineering and Imaging Sciences, King’s College London, London, UK; 5 National Heart and Lung Institute, Imperial College London, London, UK; 6 Cardiovascular MR Predevelopment, Siemens Healthcare GmbH, Erlangen, Germany; 7 Escuela de Ingeniería, Pontificia Universidad Católica de Chile, Santiago, Chile; 8 Instituto de Ingeniería Biológica y Médica, Pontificia Universidad Católica de Chile, Santiago, Chile; 9 Institute for Advanced Study, Technical University of Munich, Garching, Germany; 10 Millenium Institute for Intelligent Healthcare Engineering, Santiago, Chile

**Keywords:** Cardiac magnetic resonance angiography, Deep learning, Cardiac rest period

## Abstract

**Background:**

Coronary magnetic resonance angiography (coronary MRA) is increasingly being considered as a clinically viable method to investigate coronary artery disease (CAD). Accurate determination of the trigger delay to place the acquisition window within the quiescent part of the cardiac cycle is critical for coronary MRA in order to reduce cardiac motion. This is currently reliant on operator-led decision making, which can negatively affect consistency of scan acquisition. Recently developed deep learning (DL) derived software may overcome these issues by automation of cardiac rest period detection.

**Methods:**

Thirty individuals (female, n = 10) were investigated using a 0.9 mm isotropic image-navigator (iNAV)-based motion-corrected coronary MRA sequence. Each individual was scanned three times utilising different strategies for determination of the optimal trigger delay: (1) the DL software, (2) an experienced operator decision, and (3) a previously utilised formula for determining the trigger delay. Methodologies were compared using custom-made analysis software to assess visible coronary vessel length and coronary vessel sharpness for the entire vessel length and the first 4 cm of each vessel.

**Results:**

There was no difference in image quality between any of the methodologies for determination of the optimal trigger delay, as assessed by visible coronary vessel length, coronary vessel sharpness for each entire vessel and vessel sharpness for the first 4 cm of the left mainstem, left anterior descending or right coronary arteries. However, vessel length of the left circumflex was slightly greater using the formula method. The time taken to calculate the trigger delay was significantly lower for the DL-method as compared to the operator-led approach (106 ± 38.0 s vs 168 ± 39.2 s, p < 0.01, 95% CI of difference 25.5–98.1 s).

**Conclusions:**

Deep learning-derived automated software can effectively and efficiently determine the optimal trigger delay for acquisition of coronary MRA and thus may simplify workflow and improve reproducibility.

**Supplementary Information:**

The online version contains supplementary material available at 10.1186/s12968-023-00962-9.

## Introduction

Coronary Magnetic Resonance Angiography (coronary MRA) is being increasingly recognised as an alternative to coronary computed tomography angiography (CCTA), as it does not expose patients to either ionising radiation, or iodinated contrast. However, its clinical use has been limited by a number of factors, including suboptimal image quality, long acquisition times and the high degree of operator experience needed to obtain diagnostic image quality.

Advances in image acquisition, reconstruction and motion correction have resulted in ever decreasing scan acquisition times and improvement in image quality, as compared to the first iterations of this technology, developed 20 years ago [1, 2]. These include more efficient k-space trajectories such as 3D radial [3] or 3D variable [4–6] density spiral-like Cartesian trajectory with golden-angle rotation [7], more advanced reconstruction techniques exploiting spatiotemporal data redundancies and enabling higher acceleration factors [8, 9] as well as image-navigator (iNAV)-based non-rigid motion-corrected coronary MRA reconstruction allowing for 100% scan efficiency (i.e. no respiratory gating) [10]. Alternatively, a self-gated free-running cardiac and respiratory motion-resolved 5D whole–heart approach has been developed [11]. While the latter approach does not require scanning in the quiescent period of the cardiac cycle, with these other approaches optimal determination of the cardiac rest period is critical to reduce cardiac motion artifacts. However, determination of the optimal trigger delay and duration of the cardiac acquisition window remains very much reliant on operator input. This can increase the training requirements needed for staff to perform the scans, whilst potentially also leading to inconsistency in scan acquisition, thus impacting reproducibility. Inaccurate determination of trigger delay and acquisition window leads to increased cardiac motion during image acquisition and therefore a worsened image quality. As a result, the diagnostic accuracy is adversely impacted. In addition, this process can take 1–2 min, even with experienced operators. During this time no further scanning can occur, which is a waste of scarce CMR resources and may cause discomfort for patients. Alternatively, a formula based approach to determine the optimal trigger delay to the start of the acquisition window, previously determined by Kim et al. [12] has been developed, however this formula only determines a mid-diastolic trigger delay and cannot account for patient-specific differences in cardiac wall motion, or adapt to select the end-systolic rest period in patients who lack a sufficient mid-diastolic rest period, for example in those patients with heart rate above 65 beats per minute (bpm).

Newly developed Deep Learning (DL) software[13, 14] allows for automated determination of the mid-diastolic or end-systolic rest period, with minimal user input. This may facilitate more consistent scan acquisition, whilst simultaneously reducing the technical training required. This would also complement other recent technical developments, such as automated planning of cardiac scan acquisition and 2D iNAV [10], which track the heart during scan acquisition to ensure 100% respiratory gating efficiency.

Here we propose to investigate this DL software for cardiac rest period determination in concert with iNAV-based non-rigid motion-corrected high-resolution coronary MRA for efficient and easy-to-perform coronary MRA. The proposed approach is prospectively compared as part of a prototype iNAV-based coronary MRA scanning workflow for the first time against an operator-determined trigger delay and a formula to determine the trigger delay as the start of the acquisition window. We hypothesised that the DL software is at least as effective for trigger delay calculation as an operator led approach and a standardised formula, with a view to potentially replacing these methods within the clinical workflow.

## Methods

### Study participants

The study group consisted of 10 healthy individuals and 20 patients. Patients were recruited following their referral to CCTA to investigate possible chronic coronary syndrome (CCS). On attendance for CCTA, patients were approached and offered the opportunity to participate. Seven patients were willing and able to undergo coronary MRA within 2 h of their CCTA scan. Thirteen returned for a coronary MRA scan within 1 month. Healthy individuals were recruited following advertisement of the study at the Department of Clinical Medicine, Aarhus University Hospital. All participants provided informed written consent to participate in the study.

### Subject preparation

All individuals were asked to abstain from caffeine for 24 h prior to scanning. Patients who did not have a resting heart rate of < 60 bpm were administered 50 mg oral atenolol 2 h prior to scanning. 0.8 mg of sublingual nitroglycerine was administered to the patient group 2 min prior to commencement of the scanning in order to induce vasodilatation and improve coronary artery visualisation. Healthy volunteers were not administered beta-blockers or nitroglycerine.

### Coronary cardiovascular magnetic resonance angiography

Images were acquired using a clinical 1.5 T scanner (MAGNETOM Sola, Siemens Healthineers, Erlangen, Germany) with a 32-channel cardiac coil and an 18-channel body coil. Cardiac synchronisation was performed using a 3-lead vector electrocardiogram (ECG).

An initial thoracic localiser scan automatically identified the heart within the thoracic cavity. A single-shot two-chamber scan was followed by a modified four-chamber cine balanced steady-state free precision (bSSFP) scan perpendicular to the two-chamber scan under free—breathing. Participants were then randomised to determine the sequence of operator, formula and DL methods used to determine the optimal trigger delay for acquisition of coronary MRA to avoid bias. The 3D image volume and 2D image navigator were automatically placed.

### High resolution coronary MRA (HR-coronary MRA) image acquisition and reconstruction

The iNAV-based motion-corrected high resolution coronary MRA approach utilizing a spatial resolution of 0.9 mm^3^ has previously been described by Hajhosseiny et al. [15]. Briefly, the framework consists of an under-sampled, free-breathing 3D whole-heart ECG-triggered, bSSFP research sequence with a 3D variable density spiral-like Cartesian trajectory with golden-angle rotation. A spectrally selective spectral presaturation with inversion recovery (SPIR) pre-pulse with a constant flip angle of 130° to minimise fat-related artefact, and a T2 preparation pulse with a duration of 40 ms to improve contrast between blood and cardiac muscle, were used. Scan acquisition using the described variable density pattern was accelerated by a factor of 4.5 (nominal with respect to elliptical mask, corresponding to factor ~ 5.75 with respect to a fully sampled k-space). Additional imaging parameters included: field of view of 304 × 304 mm in coronal orientation, phase oversampling 33%, slice oversampling 25%, TE/TR 1.64/3.75 ms, flip angle 90^o.^

A 2D iNAV [10, 16] estimating beat-to-beat translational respiratory motion of the heart was used to facilitate 100% scan efficiency for all 3 methods of trigger delay calculation. The iNAV, along with the 3D image volume, was automatically placed, with review by the operator to ensure accurate placement. iNAV-based translational motion corrected data were used for reconstruction of the non-rigid motion-compensated 3D coronary MRA images as previously described [17, 18]. Reconstruction was performed in-line in the scanner software.

### Trigger delay calculation

Three different methods for determination of the trigger delay were used. Scans were acquired during either the mid-diastolic rest period or during the end systolic rest period of the cardiac cycle for the operator and automated DL assisted detection while the formula-based trigger delay detection was only used to calculate the start of the mid-diastolic rest period. A standard acquisition window length of 78 ms was used for the healthy subgroup and 10 individuals in the patient subgroup, in in order to compare the impact of the trigger delay chosen on image quality. A variable acquisition window was used in 10 patients, in order to determine the effectiveness of the DL algorithm to respond to individual patient characteristics.

### Operator identification

A single operator (GW) with 3 years of CMR experience, trained in determination of the cardiac rest period, visually reviewed the cine free—breathing 4 chamber scans to select the trigger delay. An expert operator (WYK) with 25 years of CMR experience then subsequently independently calculated trigger delay using the same scans, blinded to the original results. These results obtained by GW and WYK were compared.

### Formula

The following formula, previously derived by Kim et al. [12] was utilised for calculation of the mid-diastolic trigger delay and therefore the start of the acquisition window, on the basis of the RR interval (RR, [s]) and acquisition window (AQ, [s]):

$${T}_{d}=0.471*{RR}^{2}-0.354*RR+0.631-\left(\frac{AQ}{2}\right),$$

### Deep learning

**Fig. 1:**
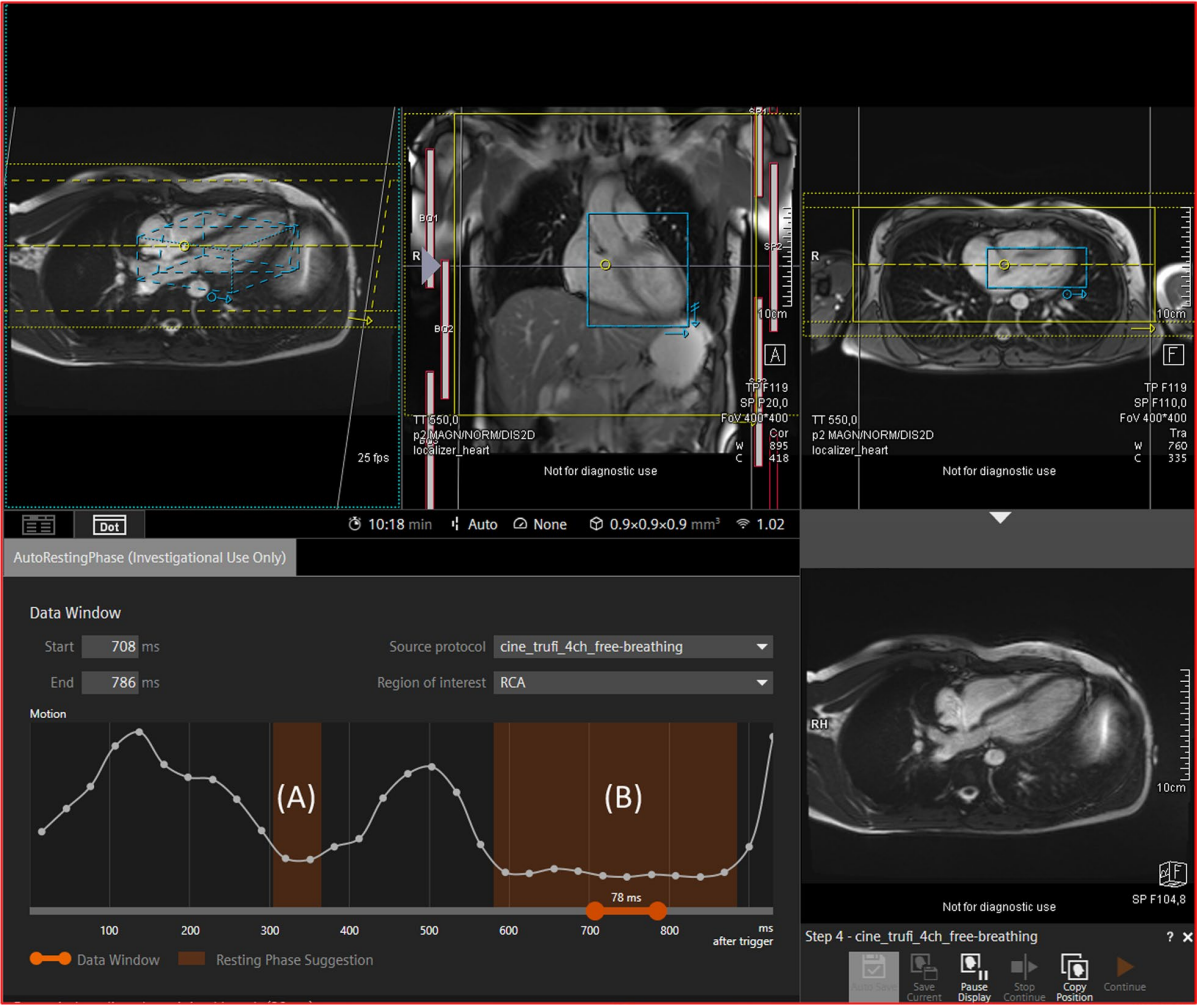
Showing the process of scan planning and the degree of automation. Following acquisition and automated analysis of a free-breathing 4-chamber scan the DL-based software identifies both the end-systolic (**A**) and mid-diastolic rest (**B**) periods, which can be used to determine the acquisition window. The DL based software also determines the position of the coronal 3D imaging slab (in yellow) and iNAV box (in blue) to be used in the coronary MRA acquisition. The placement of the shim box and 3D imaging plane does on occasion require slight manual adjustment, especially in cases where the heart is larger than average

Determination of trigger delay was performed using a research component for automatic resting phase detection, previously described in detail by Ogawa et al. [14] and Yoon et al. [13] (Siemens Healthineers, Erlangen, Germany). Briefly, the algorithm utilised a 4-chamber cine scan to quantify motion of the RCA, converted to standardised spatial and temporal size of 224 × 224 × 32, respectively. A neural network, trained on a dataset of 960 individuals, automatically identified the RCA and tracked the motion of the RCA through each frame of the cardiac cycle. The degree of motion between each frame was then calculated. The resting phase of the cardiac cycle was determined as when the motion from one frame to the next was below than the absolute threshold defined on the basis of correlation analysis with expert annotations. Following acquisition of the four-chamber cine, the right coronary artery (RCA) was localised and marked, so that the operator could ensure localisation had been performed correctly (Additional file 2: Video S1). A motion curve was then generated to graphically visualise cardiac motion. The phases of the cardiac cycle where motion was minimal were then identified either at the mid-diastolic rest period or the end systolic rest period and the appropriate trigger delay selected (Fig. 1). The operator could adjust the DL-software proposed trigger delay and acquisition window length. The latter was changed for the variable acquisition window group as appropriate according to the cardiac motion graph.

### Heart rate

Heart rate was calculated as the average heart rate throughout acquisition of coronary MRA, as calculated by CVI 42 5.13.8 (Circle Cardiovascular Imaging, Calgary, Alberta, Canada).

### Acquisition time

Coronary MRA acquisition time was automatically registered within the DICOM data and accessed from syngo.via (Siemens Healthcare GmbH).

### Time taken for calculation of trigger delay

The time taken between acquisition of the 4-chamber cine and commencement of the first coronary MRA scan was recorded, in order to determine the fastest method to calculate the trigger delay. The time of commencement of both the 4-chamber cine and the first coronary MRA was recorded as hours:minutes:seconds in syngo.via (Siemens Healthcare GmbH). The difference between these 2 timepoints was then calculated. This was calculated for the operator-led and DL methods only, as the formula method did not use the 4chamber cine to calculate trigger delay.

### Image analysis

Images were analysed by 2 independent investigators (GW and AUP) using Soap-Bubble [19] to quantitatively determine visible vessel length and vessel sharpness, as measures of image quality. The investigators were blinded to the strategy of trigger delay calculation used. Images were reformatted into a curved 2D plane, and the Left Mainstem, Left Anterior Descending (LAD), Left Circumflex (LCx) and Right Coronary (RCA) Arteries were manually tracked, quantifying visible vessel length and vessel sharpness. Vessel sharpness, calculated as the mean of the left and right edge signal intensity divided by the signal intensity at the centre of the vessel, was determined for both the first 4 cm of length of each artery, as well as the full vessel length. The mean average of the 2 investigators’ findings were taken to calculate the final results for analysis.

### Statistics

Statistical analysis was performed using GraphPad Prism version 9.4.0 and RStudio.

2023.03.1. A one sample t-test of the difference between each parameter was used to evaluate for differences between the 3 methodologies. Comparisons between the healthy and patient groups was performed using Welch's t-test. Continuous data is presented as mean ± standard deviation or mean and standard error in the case unpaired data. Ordinal data is presented as median and interquartile range. A p value of ≤ 0.05 was considered statistically significant.

## Results

### Demographics

Ten healthy individuals (female, n = 5), 10 patients with a standardised acquisition window (female, n = 4) and 10 patients with a variable acquisition window (female, n = 1) were included in the study. The patient group was significantly older (29.5 [8] vs 59.5 [45] years, p = 0.01), and had a higher BMI as compared to the healthy group (Table 1).

**Table 1:** Outlining the background demographic information of participants in the study

Demographics	n	Female	Age (years)	Height (cm)	Weight (kg)	BMI (kg/m^2^)
All	30	10	53 [52]	175.5 ± 8.6	80.1 ± 19.6	25.7 ± 4.8
Healthy	10	5	30 [8]	173.2 ± 9.8	69.3 ± 16.5	22.8 ± 3.2
Patient (all)	20	5	60 [10]	176.7 ± 7.9	85.5 ± 19.1	27.2 ± 4.9
Patient (standardised acquisition window)	10	4	61 [40]	177.0 ± 9.7	84.7 ± 21.1	26.8 ± 5.2
Patient (variable acquisition window)	10	1	59 [38]	176.0 ± 6.3	86.2 ± 18.1	27.6 ± 4.9
Healthy vs patient (all)	–	–	***0.01***	0.40	***0.03***	***0.02***
Patient (standard acquisition window) vs patient (variable acquisition window)	–	–	0.56	0.87	0.87	0.73

Age is displayed as median [IQR]. Height, weight and BMI are displayed as mean ± SD. An unpaired t-test compared differences between the healthy group and the 2 different patient groups. Statistically significant values (p <0.05) are indicated in bold

### Coronary vessel length and sharpness

**Fig. 2:**
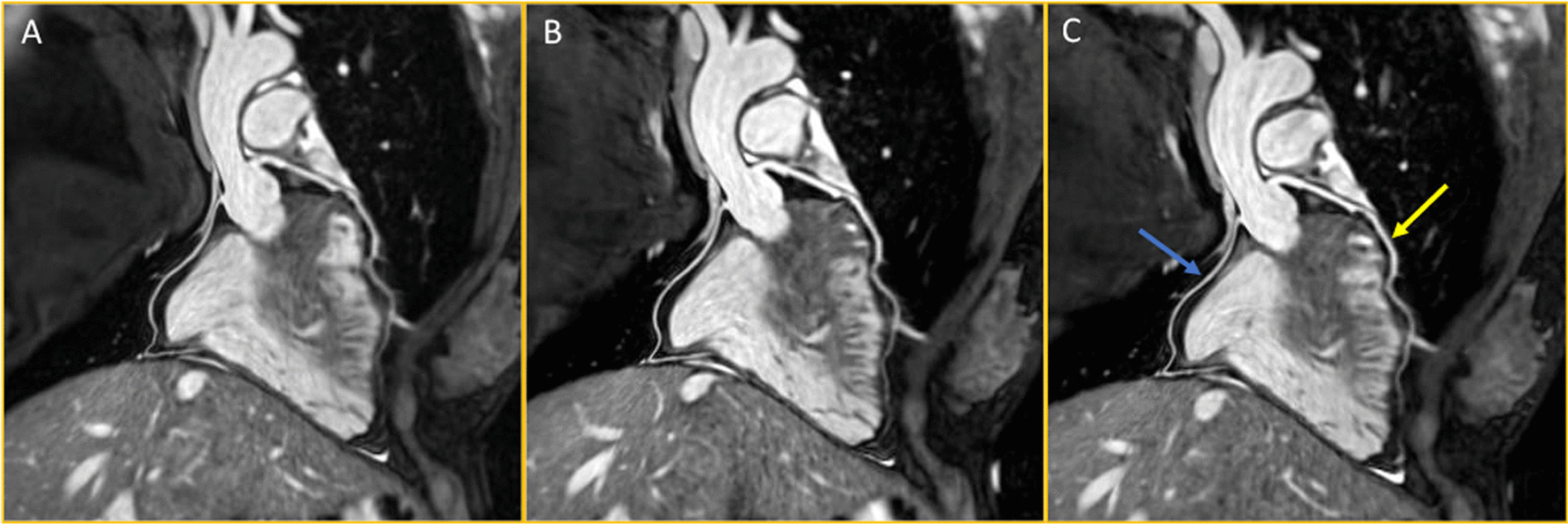
Showing reformatting of the left anterior descending artery (yellow arrow) and the right coronary artery (blue arrow) for the formula method (**A**), operator-led method (**B**) and the deep-learning method (**C**)

There was no statistically significant difference in the calculated coronary vessel length, coronary vessel sharpness along the entire vessel length, nor within the first 4 cm of each vessel for the left main, LAD or RCA. The LCx was measured as marginally shorter using the DL method as compared to the formula (Mean difference: 0.6 ± 1.4%, p = 0.02, 95% CI of difference: 0.1, 1.1 cm), however vessel sharpness did not differ between the 2 methodologies (Table 2). An example of an LAD and RCA reformat for each of the methodologies is show in Fig. 2.

**Table 2:** Showing the vessel length, vessel sharpness for the entire vessel length and vessel sharpness for the first 4 cm of each vessel for the left mainstem, left anterior descending, left circumflex and right coronary arteries

	Formula vs operator			Formula vs deep learning			Operator vs deep learning		
	Mean diff	P-value	95% CI of diff	Mean diff	P-value	95% CI of diff	Mean diff	P-value	95% CI of diff
Left main									
Vessel length (cm)	0.0 ± 0.1	0.68	− 0.0, 0.1	0.0 ± 0.2	0.80	− 0.1, 0.1	0.0 ± 0.1	0.90	− 0.1, 0.1
Vessel Sharpness (all) (%)	− 1.9 ± 6.8	0.13	− 4.5, 0.6	− 2.1 ± 8.2	0.17	− 5.2, 1.0	− 0.1 ± 6.1	0.90	− 2.4, 2.1
Left anterior descending									
Vessel length (cm)	0.3 ± 1.6	0.33	− 0.3, 0.9	0.5 ± 2.0	0.23	− 0.3, 1.2	0.2 ± 1.5	0.54	− 0.4, 0.7
Vessel sharpness (all) (%)	− 0.9 ± 3.8	0.19	− 2.4, 0.5	0.3 ± 4.5	0.69	− 1.3, 2.0	1.3 ± 4.6	0.15	− 0.5, 3.0
Vessel sharpness (first 4 cm) (%)	− 1.0 ± 3.8	0.14	− 2.5, 0.4	− 0.3 ± 4.4	0.68	− 2.0, 1.3	0.7 ± 4.3	0.37	− 0.9, 2.3
Left circumflex									
Vessel length (cm)	0.4 ± 1.5	0.19	− 0.2, 0.9	0.6 ± 1.4	***0.02***	0.1, 1.1	0.2 ± 1.3	0.32	− 0.3, 0.7
Vessel sharpness (all) (%)	0.2 ± 4.0	0.84	− 1.3, 1.6	0.6 ± 6.9	0.66	− 2.0, 3.1	0.4 ± 6.1	0.72	− 1.9, 2.7
Vessel sharpness (first 4 cm) (%)	0.1 ± 4.5	0.92	− 1.6, 1.8	0.9 ± 7.3	0.52	− 1.9, 3.6	0.8 ± 6.3	0.51	− 1.6, 3.1
Right coronary artery									
Vessel length (cm)	0.5 ± 1.7	0.10	− 0.1, 1.2	0.4 ± 2.3	0.39	− 0.5, 1.2	− 0.2 ± 2.3	0.67	− 1.0, 0.7
Vessel sharpness (all) (%)	− 0.6 ± 4.8	0.50	− 2.4, 1.2	0.7 ± 7.4	0.61	− 2.1, 3.5	1.3 ± 6.0	0.25	− 0.9, 3.5
Vessel sharpness (first 4 cm) (%)	− 0.5 ± 5.1	0.57	− 2.5, 1.4	0.2 ± 8.4	0.88	− 2.9, 3.4	0.8 ± 7.5	0.57	− 2.0, 3.6

Due to the vessel length of the left mainstem being substantially less than 4 cm, vessel sharpness for the whole vessel is displayed only. A paired t-test or a Wilcoxon Signed Rank test was used to compare differences between each method. A p value and 95% confidence interval are provided for each comparison. Data is displayed as mean ± SD. Statistically significant values (p<0.05) are indicated in bold

### Trigger delay calculation

All but two individuals were scanned during the mid-diastolic rest period for all 3 coronary MRA scans. These 2 individuals were scanned in the end-systolic rest period using the DL and operator-led methods, but during the mid-diastolic rest period using the formula.

Trigger delay from the R-wave was calculated as significantly longer in the formula as compared to the operator derived calculation (mean difference: 73 ± 99 ms, p < 0.01, 95% CI of difference: 36, 110 ms) and the DL-derived calculation (mean difference: 46 ± 101 ms, p = 0.02, 95% CI of difference: 9, 84 ms). Furthermore, there was a statistically significant difference between the operator and DL-derived methods (mean difference: 26 ± 61 ms, p = 0.02, 95% CI of difference: 4, 49 ms) (Table 3).

**Table 3:** Showing the differences in calculation of trigger delay derived from the formula, operator decision and the deep learning

Trigger delay (ms)	Formula vs operator			Formula vs deep learning			Operator vs deep learning		
	Mean diff	P-value	95% CI of diff	Mean diff	P-value	95% CI of diff	Mean diff	P-value	95% CI of diff
All	73 ± 99	** < *0.01***	36, 110	46 ± 110	***0.02***	9, 84	− 26 ± 61	***0.02***	− 49, − 4
Healthy	37 ± 93	***0.04***	2, 72	35 ± 131	0.16	− 14, 84	− 2 ± 56	0.81	− 23, 18
Patient (standardised AW)	62 ± 72	** < *0.01***	36, 89	52 ± 75	** < *0.01***	23, 80	− 11 ± 27	***0.04***	− 21, − 1
Patient (variable AW)	118 ± 110	** < *0.01***	77, 159	53 ± 87	** < *0.01***	20, 85	− 66 ± 69	** < *0.01***	− 91, − 40

	Formula			Operator			Deep learning		
	Mean diff	P-value	95% CI of diff	Mean diff	P-value	95% CI of diff	Mean diff	P-value	95% CI of diff
Healthy vs patient (all)	− 112 (35)	** < *0.01***	− 184, − 40	− 59 (50)	0.26	− 166, 48	− 95 (60)	0.14	− 224, 34
Patient (standardised AW) vs patient (variable AW)	− 65 (55)	0.26	− 183, 54	− 9 (47)	0.86	− 108, 91	− 63 (53)	0.25	− 176, 49

This is further compared between the whole cohort and the healthy and patient participants. The mean difference was analysed using a one-sample t-test and presented as mean ± SD. Unpaired data was analysed using Welch’s t test and presented as mean and standard error. AW = Acquisition Window, CI of Diff = Confidence Interval of Difference. Statistically significant values (p<0.05) are indicated in bold

Trigger delay validation showed a difference between the operator (GW) and the experienced expert (WYK) for the calculation of trigger delay (mean difference: 25 ± 33 ms, p < 0.01, 95% CI of difference: 12, 37 ms), but no difference for the duration of acquisition window (mean difference: 4 ± 21 ms, p = 0.99, 95% CI of difference: − 11, 19 ms).

### Acquisition window duration

The duration of the acquisition window calculated for the variable acquisition window subgroup was compared between each method. There was no significant difference between the formula and operator (mean difference = 3 ± 9 ms, p = 0.31, 95% CI of difference: − 9, 3 ms), the operator and DL-approaches (mean difference = 12 ± 25 ms, p = 0.18, 95% CI of difference: − 12, 25 ms) or the formula and DL methods (mean difference = 15 ± 25 ms, p = 0.10, 95% CI of difference: − 33, 3 ms).

### Acquisition time and heart rate during data acquisition

There was no difference in coronary MRA acquisition time between the formula derived, operator or DL methods (Table 4). Scan acquisition time was faster within the variable acquisition window patient subgroup as compared to the standardised acquisition window subgroup. There was a significant difference in heart rate during data acquisition between the first scan and the second and third scans performed in the whole cohort, as well as the variable acquisition window subgroup (Table 5).

**Table 4:** Showing the differences in acquisition time between the formula, operator decision and the deep learning

Acquisition time (s)	Formula vs operator			Formula vs deep learning			Operator vs deep learning		
	Mean diff	P-value	95% CI of diff	Mean diff	P-value	95% CI of diff	Mean diff	P-value	95% CI of diff
All	5 ± 59	0.68	− 18, 27	9 ± 89	0.58	− 24, 42	5 ± 57	0.66	− 17, 26
Healthy	− 15 ± 40	***0.04***	− 30, − 1	− 26 ± 66	***0.04***	− 50, − 1	− 10 ± 64	0.39	− 34, 14
Patient (standardised AW)	13 ± 19	** < *0.01***	6, 20	14 ± 25	***0.01***	4, 23	0 ± 18	0.90	− 6, 7
Patient (variable AW)	16 ± 90	0.35	− 18, 49	39 ± 129	0.11	− 9, 87	23 ± 69	0.07	− 2, 49

	Formula			Operator			Deep learning		
	Mean diff	P-value	95% CI of diff	Mean diff	P-value	95% CI of diff	mean diff	P-value	95% CI of diff
Healthy vs patient (all)	10 (54)	0.85	− 101, 121	40 (47)	0.40	− 57, 137	62 (46)	0.19	− 34, 158
Patient (standardised AW) vs patient (variable AW)	213 (50)	** < *0.01***	106, 321	216 (33)	** < *0.01***	146, 286	239 (31)	** < *0.01***	174, 303

This is further compared between the whole cohort and the healthy and patient participants. The mean difference was analysed using a one-sample t-test and presented as mean ± SD. Unpaired data was analysed using Welch’s t test and presented as mean and standard error. Statistically significant values (p<0.05) are indicated in bold

**Table 5:** Showing the differences in heart rate during the first, second and third scan acquisitions

Heart rate (bpm)	Scan 1 vs scan 2			Scan 1 vs scan 3			Scan 2 vs scan 3		
	Mean diff	P-value	95% CI of diff	Mean diff	P-value	95% CI of diff	Mean diff	P-value	95% CI of diff
All	1 ± 3	***0.01***	0, 2	1 ± 3	***0.02***	0, 3	0 ± 2	0.82	− 1, 1
Healthy	1 ± 3	0.19	0, 2	0 ± 4	0.75	− 1, 2	− 1 ± 2	0.06	− 1, 0
Patient (standardised AW)	1 ± 2	***0.04***	0, 1	1 ± 2	0.17	0, 1	− 0 ± 1	0.28	− 1, 0
Patient (variable AW)	2 ± 3	** < *0.01***	1, 3	3 ± 3	** < *0.01***	2, 5	1 ± 1	** < *0.01***	1, 2

	Scan 1			Scan 2			Scan 3		
	Mean diff	P-value	95% CI of diff	Mean diff	P-value	95% CI of diff	Mean diff	P-value	95% CI of diff
Healthy vs patient (all)	8 (4)	***0.04***	1, 16	9 (4)	***0.04***	1, 17	10 (4)	***0.02***	2, 18
Patient (standard AW) vs patient (variable AW)	2 (4)	0.67	− 6, 9	3 (3)	0.36	− 4, 10	4 (3)	0.18	− 2, 11

This is further compared between the whole cohort and the healthy and patient participants. The mean difference was analysed using a one-sample t-test and presented as mean ± SD. Unpaired data was analysed using Welch’s t test and presented as mean and standard error. Statistically significant values (p<0.05) are indicated in bold

### Time taken for calculation of trigger delay

The time taken for calculation of the trigger delay was significantly reduced using the DL software as compared to the operator (106 ± 38.0 vs 168 ± 39.2 s, p < 0.01, 95% CI of difference 25.5–98.1 s).

## Discussion

This study is the first to evaluate a recently developed DL- derived method for determination of the start of the cardiac rest period and automation of trigger delay selection for iNAV-based motion corrected coronary MRA. Our results show that this automated method was comparable to the currently used methods, as assessed by visible vessel length and image sharpness metrics. Furthermore, the time taken for calculation of trigger delay was reduced by approximately 1 min as compared to the operator led method.

The DL research software was comparable to the operator led method, which is at present the most commonly used method in coronary MRA acquisition. This was the case in all assessed metrics for both the left and right coronary vasculature, which was unsurprising given that the trigger delay calculated by both methods did not differ. Measurements of image quality and vessel length approached that found previously by Bustin et al. utilising a similar coronary MRA framework [9], albeit generally slightly reduced. Notably, there was a statistically significant difference in visible vessel length in the LCx between the formula and DL methods, however the absolute difference is minimal, and likely not of clinical relevance.

In general, most subjects with a heart rate of < 60–65 bpm have the longest cardiac rest period during end-diastole. However, when the heart rate increases, the mid-diastolic rest period becomes too short to be used, and the end-systolic rest period is usually longer. In this study only two participants were scanned during the end-systolic rest period with the DL and operator led method, however all participants were scanned in the mid diastolic period with the formula approach, as the formula was designed to calculate only the mid-diastolic rest period. These two participants account for the longer average trigger delay with the formula-based method as compared to the other 2 methods. However, since only 2 subjects had heart rates above 75 bpm that favouring the end-systolic rest period, there was no worsening of image quality in the cohort for the formula-based method. Irrespective of there being no difference in vessel sharpness or visible vessel length, the ability of the DL software to determine whether the mid-diastolic rest period is sufficient to acquire data, or whether the end-systolic rest period would be more appropriate, is a significant advantage with this new method, as it removes operator dependency and increases the speed at which this decision can be made. This software, as with both the formula and operator-led approaches, cannot, however, account for changes in heart rate once acquisition has begun, meaning that the calculated trigger delay may not be synchronised with the mid-diastolic rest period throughout the entirety of scan acquisition as it was at the commencement of scanning. This difference in heart rate is seen within this study, as the heart rate dropped significantly between scan 1 and scans 2 and 3. This is likely due to the effect of nitroglycerine causing an initial marginally increased pulse rate. Furthermore, the DL software cannot adjust for heart rate variability in individuals with arrhythmic cardiac diseases, as a regular pulse is required in order to have a consistent rest period for acquisition of coronary MRA images. However, future technological developments to reduce acquisition time, or perhaps to allow adjustment of the acquisition window during acquisition, may overcome these challenges.

The DL software also allows for patient-specific adjustment, which is not possible using the formula method. Whilst the formula method can also be rapidly calculated, the duration of the acquisition window still needs to be pre-determined and cannot be lengthened, shortened, or altered to end-systolic scanning in response to the patients’ specific rest period. Accordingly, the present study demonstrated longer trigger delay duration of the formula as compared to the operator and DL-software led approaches. Furthermore, automatic calculation and visualisation of the points in the cardiac cycle suitable for data acquisition (Fig. 1) not only allows operators to correctly select the trigger delay, but also the duration of the acquisition window, thus increasing the efficiency of scan acquisition. This is partially in accord with the present study as the acquisition time was reduced by a greater extent between the standardised and variable acquisition window patient groups using the DL software, as compared to the formula and operator methods, albeit these differences did not achieve statistical significance.

A further improvement in scan efficiency is shown by the reduction in time taken to commence coronary MRA following acquisition of the 4-chamber cine. Due to the automated nature of the trigger delay calculation, this enabled rapid placement of the acquisition window, saving approximately 1 min as compared to the operator-led approach. As such, use of the DL software appears to significantly improve the efficiency and workflow of coronary MRA.

This study also demonstrates the integration of a number of other techniques that improve the coronary MRA workflow. The automated detection of the heart within the chest cavity and the automatic placement of the 3D image volume and image navigator reduces the need for user input, as shown in Fig. 1. In total this approach increases the ease and consistency of image acquisition, allowing inexperienced operators to perform coronary MRA, and therefore facilitating the more routine use of coronary MRA.

## Limitations

There were several limitations to the study. Firstly, healthy individuals did not receive beta-blocker treatment prior to participation, in contrast to the patient group. This is due to the fact that the healthy group did not undergo CCTA scanning, and thus were not administered beta-blocker. As such the average cardiac cycle duration of the healthy group was shorter than that of the patient group. However, this did not appear to result in a significant difference in image quality (see Additional file 1). In addition, the operator could not be blinded to the trigger delay calculated by the other methods, as the same operator was responsible for control of the MR scanner. However, the operator was blinded to the methods used during data analysis and an expert, blinded to the original results, retrospectively validated the operator-led trigger delay calculations. The difference in trigger delay calculation was statistically significant, however a mean difference of 25 ms is minimal and unlikely to have a clinical relevance. The ratio of male to female participants within the patient subgroups was uneven, meaning that women are under-represented in this study. Although this may theoretically introduce bias, to our knowledge there are no specific differences in physiology between sexes that could affect the results of this study. Finally, the DL-derived method was tested within a narrow range of heart rates, with a mean of approximately 60 bpm. However, a heart rate of 60 bpm or lower would also be the recommended heart rate for coronary MRA.

## Conclusions

DL-derived automated detection of optimal cardiac trigger delay and automated acquisition planning performs similar to an operator determined and a mathematical formula for selection of the optimal motion free acquisition window for iNAV-based motion-corrected coronary MRA, whilst reducing time taken to calculate trigger delay and placement of the acquisition window. It can, therefore, be routinely introduced into the coronary MRA protocol, standardising workflow, and improving efficiency of iNAV-based motion-corrected coronary MRA acquisition.

### Supplementary Information

**Additional file 1: Table S1**. Table showing the results for vessel length and vessels sharpness for the left main stem for the different methods of trigger delay calculation as an entire cohort and in healthy and patient subgroups. The mean difference was analysed using a one-sample t-test and presented as mean ± SD . Unpaired data was analysed using Welch’s t test and presented as mean and standard error. Statistically significant values (p < 0.05) are indicated in bold. **Table S2**: Table showing the results for vessel length and vessels sharpness for the LAD for the different methods of trigger delay calculation as an entire cohort and in healthy and patient subgroups. The mean difference was analysed using a one-sample t-test and presented as mean ± SD . Unpaired data was analysed using Welch’s t test and presented as mean and standard error. Statistically significant values (p<0.05) are indicated in bold. **Table S3**: Table showing the results for vessel length and vessels sharpness for the LCX for the different methods of trigger delay calculation as an entire cohort and in healthy and patient subgroups. The mean difference was analysed using a one-sample t-test and presented as mean ± SD. Unpaired data was analysed using Welch’s t test and presented as mean and standard error. Statistically significant values (p<0.05) are indicated in bold. **Table S4**: Table showing the results for vessel length and vessels sharpness for the RCA for the different methods of trigger delay calculation as an entire cohort and in healthy and patient subgroups. The mean difference was analysed using a one-sample t-test and presented as mean ± SD. Unpaired data was analysed using Welch’s t test and presented as mean and standard error. Statistically significant values (p<0.05) are indicated in bold.

**Additional file 2: Video S1.** Video showing A) video of the free-breathing 4 chamber cine scan used for right coronary artery (RCA) tracking to determine cardiac motion and B) overlay of the same image to illustrate the motion of the RCA.

## Data Availability

The datasets used and/or analysed during the current study are available from the corresponding author on reasonable request.

## Abbreviations

AQ: Acquisition window
bSSFP: Balanced steady-state free precision
BPM: Beats per minute
CAD: Coronary artery disease
CCS: Chronic coronary syndrome
CCTA: Coronary computer tomography angiography
Coronary MRA: Coronary magnetic resonance angiography
DL: Deep learning
ECG: Electrocardiogram
iNAV: Image navigator
LAD: Left anterior descending artery
LCX: Left circumflex artery
RCA: Right coronary artery
RR: R–R interval
SPIR: Spectral presaturation with inversion recovery
