# Supplementary material for: Automated detection of cardiac rest period for trigger delay calculation for image-based navigator coronary magnetic resonance angiography
Source: J Cardiovasc Magn Reson. 2023 Oct 2;25:52. doi: 10.1186/s12968-023-00962-9 (PMC10544388; doi:10.1186/s12968-023-00962-9)
Supplement: Supplementary file 1 — Additional file 1: Table S1. Table showing the results for vessel length and vessels sharpness for the left main stem for the different methods of trigger delay calculation as an entire cohort and in healthy and patient subgroups. The mean difference was analysed using a one-sample t-test and presented as mean ± SD . Unpaired data was analysed using Welch’s t test and presented as mean and standard error. Statistically significant values (p < 0.05) are indicated in bold. Table S2: Table showing the results for vessel length and vessels sharpness for the LAD for the different methods of trigger delay calculation as an entire cohort and in healthy and patient subgroups. The mean difference was analysed using a one-sample t-test and presented as mean ± SD . Unpaired data was analysed using Welch’s t test and presented as mean and standard error. Statistically significant values (p<0.05) are indicated in bold. Table S3: Table showing the results for vessel length and vessels sharpness for the LCX for the different methods of trigger delay calculation as an entire cohort and in healthy and patient subgroups. The mean difference was analysed using a one-sample t-test and presented as mean ± SD. Unpaired data was analysed using Welch’s t test and presented as mean and standard error. Statistically significant values (p<0.05) are indicated in bold. Table S4: Table showing the results for vessel length and vessels sharpness for the RCA for the different methods of trigger delay calculation as an entire cohort and in healthy and patient subgroups. The mean difference was analysed using a one-sample t-test and presented as mean ± SD. Unpaired data was analysed using Welch’s t test and presented as mean and standard error. Statistically significant values (p<0.05) are indicated in bold. [file 12968_2023_962_MOESM1_ESM.docx]

Additional file 1 Data

| **Left Main** | Formula vs Operator | | | Formula vs Deep Learning | | | Operator vs Deep Learning | | |
| --- | --- | --- | --- | --- | --- | --- | --- | --- | --- |
| **Vessel Length (cm)** | Mean Diff. | P-value | 95% CI of Diff. | Mean Diff. | P-value | 95% CI of Diff. | Mean Diff. | P-value | 95% CI of Diff. |
| *All* | 0.01 ± 0.14 | 0.68 | -0.04, 0.06 | 0.01 ± 0.17 | 0.80 | -0.05, 0.07 | 0.00 ± 0.14 | 0.90 | -0.06, 0.05 |
| *Healthy* | -0.04 ± 0.11 | 0.05 | -0.08, 0.00 | -0.07 ± 0.12 | ***<0.01*** | -0.11, -0.02 | -0.03 ± 0.12 | 0.22 | -0.07, 0.02 |
| *Patient (All)* | 0.04 ± 0.15 | 0.28 | -0.03, 0.11 | 0.05 ± 0.17 | 0.26 | -0.04, 0.13 | 0.01 ± 0.15 | 0.80 | -0.06, 0.08 |
| *Patient (Standardised AW)* | 0.09 ± 0.12 | ***<0.01*** | 0.05, 0.14 | 0.09 ± 0.22 | ***0.04*** | 0.00, 0.17 | -0.01 ± 0.13 | 0.82 | -0.05, 0.04 |
| *Patient (Variable AW)* | -0.02 ± 0.15 | 0.50 | -0.07, 0.04 | 0.00 ± 0.08 | 0.79 | -0.03, 0.03 | 0.02 ± 0.16 | 0.46 | -0.04, 0.08 |
|  | Formula | | | Operator | | | Deep Learning | | |
|  | Mean Diff. | P-value | 95% CI of Diff. | Mean Diff. | P-value | 95% CI of Diff. | Mean Diff. | P-value | 95% CI of Diff. |
| *Healthy vs Patient (All)* | -0.18 (0.15) | 0.24 | -0.49, 0.13 | -0.10 (0.15) | 0.50 | -0.42, 0.21 | -0.07 (0.15) | 0.66 | -0.38, 0.24 |
| *Patient (Standardised AW) Vs Patient (Variable AW)* | 0.17 (0.22) | 0.44 | -0.29, 0.64 | 0.06 (0.19) | 0.74 | -0.33, 0.46 | 0.09 (0.19) | 0.64 | -0.31, 0.49 |
| **Vessel Sharpness All (%)** | Formula vs Operator | | | Formula vs Deep Learning | | | Operator vs Deep Learning | | |
|  | Mean Diff. | P-value | 95% CI of Diff. | Mean Diff. | P-value | 95% CI of Diff. | Mean Diff. | P-value | 95% CI of Diff. |
| *All* | -1.94 ± 6.84 | 0.13 | -4.50, 0.61 | -2.09 ± 8.19 | 0.17 | -5.15, 0.97 | -0.14 ± 6.07 | 0.90 | -2.41, 2.12 |
| *Healthy* | 0.17 ± 4.73 | 0.85 | -1.60, 1.93 | -1.45 ± 5.41 | 0.15 | -3.46, 0.57 | -1.61 ± 6.40 | 0.18 | -4.00, 0.78 |
| *Patient (All)* | -3.00 ± 7.51 | 0.09 | -6.51, 0.52 | -2.41 ± 9.34 | 0.26 | -6.78, 1.96 | 0.59 ± 5.80 | 0.66 | -2.13, 3.30 |
| *Patient (Standardised AW)* | -0.67 ± 3.32 | 0.27 | -1.91, 0.56 | -2.22 ± 5.33 | ***0.03*** | -4.21, -0.22 | -1.54 ± 5.81 | 0.16 | -3.71, 0.63 |
| *Patient (Variable AW)* | -5.32 ± 9.42 | ***<0.01*** | -8.84, -1.80 | -2.60 ± 11.95 | 0.24 | -7.06, 1.86 | 2.72 ± 4.80 | ***<0.01*** | 0.93, 4.51 |
|  | Formula | | | Operator | | | Deep Learning | | |
|  | Mean Diff. | P-value | 95% CI of Diff. | Mean Diff. | P-value | 95% CI of Diff. | Mean Diff. | P-value | 95% CI of Diff. |
| *Healthy vs Patient (All)* | 2.63 (3.82) | 0.50 | -5.32, 10.58 | -0.53 (3.74) | 0.89 | -8.54, 7.48 | 1.67 (3.14) | 0.60 | -4.93, 8.26 |
| *Patient (Standardised AW) Vs Patient (Variable AW)* | -1.08 (4.99) | 0.83 | -11.58, 9.43 | -5.72 (3.31) | 0.11 | -12.94, 1.49 | -1.46 (3.7) | 0.70 | -9.67, 6.75 |

Table S1: Table showing the results for vessel length and vessels sharpness for the left main stem for the different methods of trigger delay calculation as an entire cohort and in healthy and patient subgroups. The mean difference was analysed using a one-sample t-test and presented as mean ± SD . Unpaired data was analysed using Welch’s t test and presented as mean and standard error.

Table S2: Table showing the results for vessel length and vessels sharpness for the LAD for the different methods of trigger delay calculation as an entire cohort and in healthy and patient subgroups. The mean difference was analysed using a one-sample t-test and presented as mean ± SD . Unpaired data was analysed using Welch’s t test and presented as mean and standard error.

| **Left Anterior Descending (LAD)** | Formula vs Operator | | | Formula vs Deep Learning | | | Operator vs Deep Learning | | |
| --- | --- | --- | --- | --- | --- | --- | --- | --- | --- |
| **Vessel Length (cm)** | Mean Diff. | P-value | 95% CI of Diff. | Mean Diff. | P-value | 95% CI of Diff. | Mean Diff. | P-value | 95% CI of Diff. |
| *All* | 0.29 ± 1.59 | 0.33 | -0.31, 0.88 | 0.45 ± 2.03 | 0.23 | -0.31, 1.21 | 0.16 ± 1.46 | 0.54 | -0.38, 0.71 |
| *Healthy* | -0.56 ± 1.27 | ***0.02*** | -1.03, -0.09 | -0.32 ± 1.48 | 0.25 | -0.87, 0.24 | 0.24 ± 0.63 | ***0.04*** | 0.01, 0.48 |
| *Patient (All)* | 0.72 ± 1.58 | 0.06 | -0.02, 1.45 | 0.84 ± 2.17 | 0.10 | -0.18, 1.86 | 0.12 ± 1.75 | 0.76 | -0.70, 0.94 |
| *Patient (Standardised AW)* | 0.40 ± 1.25 | 0.09 | -0.07, 0.86 | 0.88 ± 2.42 | 0.06 | -0.02, 1.78 | 0.48 ± 1.94 | 0.19 | -0.24, 1.21 |
| *Patient (Variable AW)* | 1.03 ± 1.77 | ***<0.01*** | 0.37, 1.69 | 0.80 ± 1.85 | ***0.03*** | 0.11, 1.49 | -0.23 ± 1.41 | 0.37 | -0.76, 0.29 |
|  | Formula | | | Operator | | | Deep Learning | | |
|  | Mean Diff. | P-value | 95% CI of Diff. | Mean Diff. | P-value | 95% CI of Diff. | Mean Diff. | P-value | 95% CI of Diff. |
| *Healthy vs Patient (All)* | -0.21 (1.11) | 0.85 | -2.55, 2.12 | 1.06 (1.05) | 0.33 | -1.16, 3.29 | 0.94 (1.08) | 0.39 | -1.31, 3.19 |
| *Patient (Standardised AW) Vs Patient (Variable AW)* | -0.10 (1.34) | 0.94 | -2.95, 2.76 | 0.54 (1.13) | 0.64 | -1.84, 2.92 | -0.17 (1.33) | 0.9 | -2.97, 2.62 |
| **Vessel Sharpness (All) (%)** | Formula vs Operator | | | Formula vs Deep Learning | | | Operator vs Deep Learning | | |
|  | Mean Diff. | P-value | 95% CI of Diff. | Mean Diff. | P-value | 95% CI of Diff. | Mean Diff. | P-value | 95% CI of Diff. |
| *All* | -0.93 ± 3.80 | 0.19 | -2.35, 0.49 | 0.32 ± 4.45 | 0.69 | -1.34, 1.99 | 1.25 ± 4.61 | 0.15 | -0.47, 2.97 |
| *Healthy* | -0.43 ± 4.21 | 0.58 | -2.00, 1.14 | 0.50 ± 4.83 | 0.58 | -1.31, 2.30 | 0.93 ± 4.62 | 0.28 | -0.80, 2.65 |
| *Patient (All)* | -1.18 ± 3.59 | 0.16 | -2.86, 0.50 | 0.24 ± 4.29 | 0.81 | -1.77, 2.24 | 1.42 ± 4.64 | 0.19 | -0.75, 3.59 |
| *Patient (Standardised AW)* | -1.15 ± 2.63 | ***0.02*** | -2.13, -0.16 | -0.95 ± 4.08 | 0.21 | -2.48, 0.57 | 0.20 ± 4.88 | 0.83 | -1.63, 2.02 |
| *Patient (Variable AW)* | -1.21 ± 4.28 | 0.13 | -2.81, 0.39 | 1.43 ± 4.06 | 0.06 | -0.09, 2.94 | 2.64 ± 3.92 | ***<0.01*** | 1.17, 4.10 |
|  | Formula | | | Operator | | | Deep Learning | | |
|  | Mean Diff. | P-value | 95% CI of Diff. | Mean Diff. | P-value | 95% CI of Diff. | Mean Diff. | P-value | 95% CI of Diff. |
| *Healthy vs Patient (All)* | 1.76 (2.97) | 0.56 | -4.55, 8.06 | 1.01 (3.17) | 0.75 | -5.76, 7.78 | 1.50 (2.68) | 0.58 | -4.16, 7.16 |
| *Patient (Standardised AW) Vs Patient (Variable AW)* | -4.15 (2.93) | 0.17 | -10.32, 2.02 | -4.21 (2.91) | 0.17 | -10.36, 1.93 | -1.77 (3.02) | 0.56 | -8.14, 4.59 |
| **Vessel Sharpness (First 4cm) (%)** | Formula vs Operator | | | Formula vs Deep Learning | | | Operator vs Deep Learning | | |
|  | Mean Diff. | P-value | 95% CI of Diff. | Mean Diff. | P-value | 95% CI of Diff. | Mean Diff. | P-value | 95% CI of Diff. |
| *All* | -1.04 ± 3.82 | 0.14 | -2.47, 0.38 | -0.34 ± 4.44 | 0.68 | -2.00, 1.32 | 0.70 ± 4.25 | 0.37 | -0.88, 2.29 |
| *Healthy* | -1.58 ± 4.80 | 0.08 | -3.37, 0.22 | -0.88 ± 4.90 | 0.33 | -2.71, 0.95 | 0.70 ± 4.24 | 0.38 | -0.89, 2.28 |
| *Patient (All)* | -0.78 ± 3.21 | 0.29 | -2.28, 0.72 | -0.07 ± 4.21 | 0.94 | -2.04, 1.90 | 0.71 ± 4.29 | 0.47 | -1.30, 2.72 |
| *Patient (Standardised AW)* | -0.67 ± 2.66 | 0.18 | -1.67, 0.32 | -1.31 ± 3.73 | 0.06 | -2.71, 0.08 | -0.64 ± 3.14 | 0.28 | -1.81, 0.54 |
| *Patient (Variable AW)* | -0.88 ± 3.62 | 0.19 | -2.23, 0.47 | 1.18 ± 4.20 | 0.14 | -0.39, 2.74 | 2.06 ± 4.74 | ***0.02*** | 0.28, 3.83 |
|  | Formula | | | Operator | | | Deep Learning | | |
|  | Mean Diff. | P-value | 95% CI of Diff. | Mean Diff. | P-value | 95% CI of Diff. | Mean Diff. | P-value | 95% CI of Diff. |
| *Healthy vs Patient (All)* | -0.99 (3.24) | 0.77 | -7.96, 5.98 | -0.19 (3.35) | 0.96 | -7.40, 7.03 | -0.17 (3.03) | 0.95 | -6.59, 6.24 |
| *Patient (Standardised AW) Vs Patient (Variable AW)* | -4.54 (2.78) | 0.12 | -10.39, 1.31 | -4.74 (2.83) | 0.11 | -10.71, 1.22 | -2.05 (3.25) | 0.54 | -8.89, 4.79 |

Table S3: Table showing the results for vessel length and vessels sharpness for the LCX for the different methods of trigger delay calculation as an entire cohort and in healthy and patient subgroups. The mean difference was analysed using a one-sample t-test and presented as mean ± SD . Unpaired data was analysed using Welch’s t test and presented as mean and standard error.

| **Left Circumflex (LCx)** | Formula vs Operator | | | Formula vs Deep Learning | | | Operator vs Deep Learning | | |
| --- | --- | --- | --- | --- | --- | --- | --- | --- | --- |
| **Vessel Length (cm)** | Mean Diff. | P-value | 95% CI of Diff. | Mean Diff. | P-value | 95% CI of Diff. | Mean Diff. | P-value | 95% CI of Diff. |
| *All* | 0.35 ± 1.46 | 0.19 | -0.19, 0.90 | 0.59 ± 1.35 | ***0.02*** | 0.09, 1.10 | 0.24 ± 1.30 | 0.32 | -0.25, 0.72 |
| *Healthy* | -0.22 ± 0.77 | 0.13 | -0.51, 0.07 | 0.28 ± 0.92 | 0.11 | -0.06, 0.62 | 0.50 ± 0.96 | ***0.01*** | 0.14, 0.86 |
| *Patient (All)* | 0.64 ± 1.64 | 0.10 | -0.13, 1.41 | 0.75 ± 1.50 | ***0.04*** | 0.05, 1.45 | 0.11 ± 1.43 | 0.74 | -0.56, 0.78 |
| *Patient (Standardised AW)* | 0.49 ± 1.00 | ***0.01*** | 0.12, 0.87 | 0.95 ± 1.57 | ***0.00*** | 0.36, 1.54 | 0.46 ± 0.95 | ***0.01*** | 0.10, 0.81 |
| *Patient (Variable AW)* | 0.79 ± 2.06 | 0.04 | 0.02, 1.56 | 0.55 ± 1.37 | ***0.04*** | 0.03, 1.06 | -0.24 ± 1.69 | 0.44 | -0.88, 0.39 |
|  | Formula | | | Operator | | | Deep Learning | | |
|  | Mean Diff. | P-value | 95% CI of Diff. | Mean Diff. | P-value | 95% CI of Diff. | Mean Diff. | P-value | 95% CI of Diff. |
| *Healthy vs Patient (All)* | -1.63 (0.91) | 0.08 | -3.49, 0.23 | -0.76 (0.81) | 0.36 | -2.44, 0.91 | -1.16 (0.88) | 0.20 | -2.97, 0.66 |
| *Patient (Standardised AW) Vs Patient (Variable AW)* | -0.86 (1.51) | 0.58 | -4.13, 2.42 | -0.56 (1.23) | 0.66 | -3.24, 2.12 | -1.26 (1.24) | 0.33 | -4.02, 1.49 |
| **Vessel Sharpness (All) (%)** | Formula vs Operator | | | Formula vs Deep Learning | | | Operator vs Deep Learning | | |
|  | Mean Diff. | P-value | 95% CI of Diff. | Mean Diff. | P-value | 95% CI of Diff. | Mean Diff. | P-value | 95% CI of Diff. |
| *All* | 0.15 ± 3.96 | 0.84 | -1.33, 1.63 | 0.55 ± 6.91 | 0.66 | -2.03, 3.13 | 0.40 ± 6.12 | 0.72 | -1.88, 2.69 |
| *Healthy* | -0.70 ± 2.70 | 0.17 | -1.71, 0.31 | 1.68 ± 9.88 | 0.36 | -2.00, 5.37 | 2.38 ± 8.97 | 0.16 | -0.97, 5.74 |
| *Patient (All)* | 0.58 ± 4.43 | 0.57 | -1.49, 2.65 | -0.01 ± 4.72 | 0.99 | -2.22, 2.19 | -0.59 ± 3.62 | 0.47 | -2.29, 1.10 |
| *Patient (Standardised AW)* | 0.23 ± 5.72 | 0.82 | -1.90, 2.37 | 0.15 ± 5.89 | 0.89 | -2.05, 2.35 | -0.09 ± 3.70 | 0.90 | -1.47, 1.29 |
| *Patient (Variable AW)* | 0.92 ± 2.36 | ***0.04*** | 0.04, 1.80 | -0.17 ± 2.99 | 0.75 | -1.29, 0.94 | -1.09 ± 3.41 | 0.09 | -2.37, 0.18 |
|  | Formula | | | Operator | | | Deep Learning | | |
|  | Mean Diff. | P-value | 95% CI of Diff. | Mean Diff. | P-value | 95% CI of Diff. | Mean Diff. | P-value | 95% CI of Diff. |
| *Healthy vs Patient (All)* | 1.68 (2.65) | 0.53 | -3.92, 7.29 | 2.96 (2.78) | 0.30 | -2.89, 8.81 | -0.01 (3.91) | 1.00 | -8.48, 8.45 |
| *Patient (Standardised AW) Vs Patient (Variable AW)* | -5.14 (2.73) | 0.08 | -10.98, 0.69 | -4.46 (3.13) | 0.18 | -11.27, 2.36 | -5.46 (3.02) | 0.09 | -11.98, 1.06 |
| **Vessel Sharpness (First 4cm) (%)** | Formula vs Operator | | | Formula vs Deep Learning | | | Operator vs Deep Learning | | |
|  | Mean Diff. | P-value | 95% CI of Diff. | Mean Diff. | P-value | 95% CI of Diff. | Mean Diff. | P-value | 95% CI of Diff. |
| *All* | 0.08 ± 4.50 | 0.92 | -1.60, 1.77 | 0.86 ± 7.30 | 0.52 | -1.87, 3.59 | 0.77 ± 6.31 | 0.51 | -1.58, 3.13 |
| *Healthy* | -0.92 ± 3.50 | 0.16 | -2.23, 0.39 | 2.01 ± 9.83 | 0.27 | -1.66, 5.68 | 2.93 ± 8.73 | 0.08 | -0.33, 6.19 |
| *Patient (All)* | 0.59 ± 4.89 | 0.60 | -1.70, 2.88 | 0.28 ± 5.59 | 0.82 | -2.33, 2.90 | -0.30 ± 4.29 | 0.76 | -2.31, 1.71 |
| *Patient (Standardised AW)* | 0.64 ± 5.98 | 0.56 | -1.60, 2.87 | 0.93 ± 7.13 | 0.48 | -1.73, 3.59 | 0.30 ± 4.53 | 0.72 | -1.39, 1.99 |
| *Patient (Variable AW)* | 0.54 ± 3.35 | 0.39 | -0.71, 1.79 | -0.36 ± 3.12 | 0.53 | -1.53, 0.80 | -0.90 ± 3.88 | 0.21 | -2.35, 0.55 |
|  | Formula | | | Operator | | | Deep Learning | | |
|  | Mean Diff. | P-value | 95% CI of Diff. | Mean Diff. | P-value | 95% CI of Diff. | Mean Diff. | P-value | 95% CI of Diff. |
| *Healthy vs Patient (All)* | 1.28 (2.71) | 0.64 | -4.46, 7.02 | 2.79 (2.82) | 0.34 | -3.15, 8.73 | -0.44 (3.93) | 0.91 | -8.95, 8.07 |
| *Patient (Standardised AW) Vs Patient (Variable AW)* | -3.95 (2.75) | 0.17 | -9.81, 1.91 | -4.05 (3.14) | 0.22 | -10.78, 2.68 | -5.25 (3.09) | 0.11 | -11.91, 1.41 |

Table S4: Table showing the results for vessel length and vessels sharpness for the RCA for the different methods of trigger delay calculation as an entire cohort and in healthy and patient subgroups. The mean difference was analysed using a one-sample t-test and presented as mean ± SD . Unpaired data was analysed using Welch’s t test and presented as mean and standard error.

| **Right Coronary Artery (RCA)** | Formula vs Operator | | | Formula vs Deep Learning | | | Operator vs Deep Learning | | |
| --- | --- | --- | --- | --- | --- | --- | --- | --- | --- |
| **Vessel Length (cm)** | Mean Diff. | P-value | 95% CI of Diff. | Mean Diff. | P-value | 95% CI of Diff. | Mean Diff. | P-value | 95% CI of Diff. |
| *All* | 0.54 ± 1.70 | 0.10 | -0.10, 1.17 | 0.36 ± 2.25 | 0.39 | -0.48, 1.20 | -0.18 ± 2.26 | 0.67 | -1.02, 0.67 |
| *Healthy* | 0.28 ± 1.51 | 0.32 | -0.28, 0.85 | 0.75 ± 2.03 | 0.05 | -0.01, 1.51 | 0.47 ± 2.64 | 0.34 | -0.51, 1.46 |
| *Patient (All)* | 0.66 ± 1.79 | 0.11 | -0.17, 1.50 | 0.16 ± 2.35 | 0.76 | -0.94, 1.26 | -0.50 ± 1.97 | 0.27 | -1.43, 0.42 |
| *Patient (Standardised AW)* | 0.44 ± 1.36 | 0.09 | -0.07, 0.95 | -0.22 ± 0.85 | 0.17 | -0.53, 0.10 | -0.65 ± 1.63 | ***0.04*** | -1.26, -0.05 |
| *Patient (Variable AW)* | 0.89 ± 2.08 | ***0.03*** | 0.11, 1.66 | 0.53 ± 3.13 | 0.36 | -0.64, 1.70 | -0.35 ± 2.22 | 0.39 | -1.18, 0.48 |
|  | Formula | | | Operator | | | Deep Learning | | |
|  | Mean Diff. | P-value | 95% CI of Diff. | Mean Diff. | P-value | 95% CI of Diff. | Mean Diff. | P-value | 95% CI of Diff. |
| *Healthy vs Patient (All)* | -0.35 (1.20) | 0.77 | -2.85, 2.15 | 0.03 (1.41) | 0.98 | -2.93, 2.98 | -0.95 (1.33) | 0.48 | -3.72, 1.83 |
| *Patient (Standardised AW) Vs Patient (Variable AW)* | -2.20 (1.53) | 0.17 | -5.44, 1.03 | -1.75 (1.67) | 0.31 | -5.29, 1.78 | -1.45 (1.61) | 0.38 | -4.87, 1.97 |
| **Vessel Sharpness (All) (%)** | Formula vs Operator | | | Formula vs Deep Learning | | | Operator vs Deep Learning | | |
|  | Mean Diff. | P-value | 95% CI of Diff. | Mean Diff. | P-value | 95% CI of Diff. | Mean Diff. | P-value | 95% CI of Diff. |
| *All* | -0.60 ± 4.84 | 0.50 | -2.41, 1.21 | 0.69 ± 7.39 | 0.61 | -2.07, 3.45 | 1.29 ± 5.96 | 0.25 | -0.94, 3.51 |
| *Healthy* | -1.22 ± 4.70 | 0.17 | -2.97, 0.53 | 1.00 ± 11.44 | 0.64 | -3.27, 5.27 | 2.22 ± 8.55 | 0.17 | -0.97, 5.42 |
| *Patient (All)* | -0.29 ± 4.92 | 0.80 | -2.59, 2.01 | 0.53 ± 4.10 | 0.57 | -1.39, 2.45 | 0.82 ± 4.04 | 0.38 | -1.07, 2.71 |
| *Patient (Standardised AW)* | 0.15 ± 5.29 | 0.88 | -1.83, 2.13 | 0.37 ± 4.38 | 0.64 | -1.26, 2.01 | 0.22 ± 4.07 | 0.77 | -1.30, 1.74 |
| *Patient (Variable AW)* | -0.73 ± 4.37 | 0.37 | -2.36, 0.91 | 0.69 ± 3.71 | 0.32 | -0.70, 2.07 | 1.41 ± 3.83 | 0.05 | -0.02, 2.85 |
|  | Formula | | | Operator | | | Deep Learning | | |
|  | Mean Diff. | P-value | 95% CI of Diff. | Mean Diff. | P-value | 95% CI of Diff. | Mean Diff. | P-value | 95% CI of Diff. |
| *Healthy vs Patient (All)* | 1.60 (3.31) | 0.63 | -5.34, 8.54 | 2.53 (2.93) | 0.40 | -3.49, 8.56 | 1.13 (3.70) | 0.76 | -6.69, 8.95 |
| *Patient (Standardised AW) Vs Patient (Variable AW)* | -2.96 (4.00) | 0.47 | -11.37, 5.44 | -3.84 (4.23) | 0.38 | -12.84, 5.16 | -2.65 (4.02) | 0.52 | -11.19, 5.89 |
| **Vessel Sharpness (First 4cm) (%)** | Formula vs Operator | | | Formula vs Deep Learning | | | Operator vs Deep Learning | | |
|  | Mean Diff. | P-value | 95% CI of Diff. | Mean Diff. | P-value | 95% CI of Diff. | Mean Diff. | P-value | 95% CI of Diff. |
| *All* | -0.54 ± 5.14 | 0.57 | -2.46, 1.38 | 0.24 ± 8.44 | 0.88 | -2.91, 3.39 | 0.78 ± 7.48 | 0.57 | -2.01, 3.57 |
| *Healthy* | 0.88 ± 4.68 | 0.31 | -0.87, 2.63 | 1.84 ± 13.33 | 0.46 | -3.14, 6.81 | 0.96 ± 11.46 | 0.65 | -3.32, 5.24 |
| *Patient (All)* | -1.25 ± 5.25 | 0.30 | -3.70, 1.21 | -0.55 ± 4.04 | 0.55 | -2.45, 1.34 | 0.69 ± 4.30 | 0.48 | -1.32, 2.70 |
| *Patient (Standardised AW)* | -0.87 ± 6.22 | 0.45 | -3.19, 1.45 | -0.39 ± 5.01 | 0.67 | -2.26, 1.48 | 0.48 ± 4.97 | 0.60 | -1.38, 2.33 |
| *Patient (Variable AW)* | -1.62 ± 3.91 | ***0.03*** | -3.08, -0.16 | -0.72 ± 2.64 | 0.15 | -1.70, 0.26 | 0.90 ± 3.40 | 0.16 | -0.37, 2.17 |
|  | Formula | | | Operator | | | Deep Learning | | |
|  | Mean Diff. | P-value | 95% CI of Diff. | Mean Diff. | P-value | 95% CI of Diff. | Mean Diff. | P-value | 95% CI of Diff. |
| *Healthy vs Patient (All)* | 4.92 (3.66) | 0.19 | -2.76, 12.60 | 2.80 (3.24) | 0.40 | -3.87, 9.47 | 2.53 (4.23) | 0.56 | -6.43, 11.49 |
| *Patient (Standardised AW) Vs Patient (Variable AW)* | -4.46 (4.23) | 0.31 | -13.47, 4.54 | -5.22 (4.76) | 0.30 | -15.62, 5.19 | -4.79 (4.40) | 0.29 | -14.24, 4.65 |

s
