# Supplementary material for: Automated detection of cardiac rest period for trigger delay calculation for image-based navigator coronary magnetic resonance angiography
Source: J Cardiovasc Magn Reson. 2023 Oct 2;25:52. doi: 10.1186/s12968-023-00962-9 (PMC10544388; doi:10.1186/s12968-023-00962-9)
Supplement: Supplementary file 2 — Additional file 2: Video S1. Video showing A) video of the free-breathing 4 chamber cine scan used for right coronary artery (RCA) tracking to determine cardiac motion and B) overlay of the same image to illustrate the motion of the RCA. [file 12968_2023_962_MOESM2_ESM.pptx]

## Slide 1
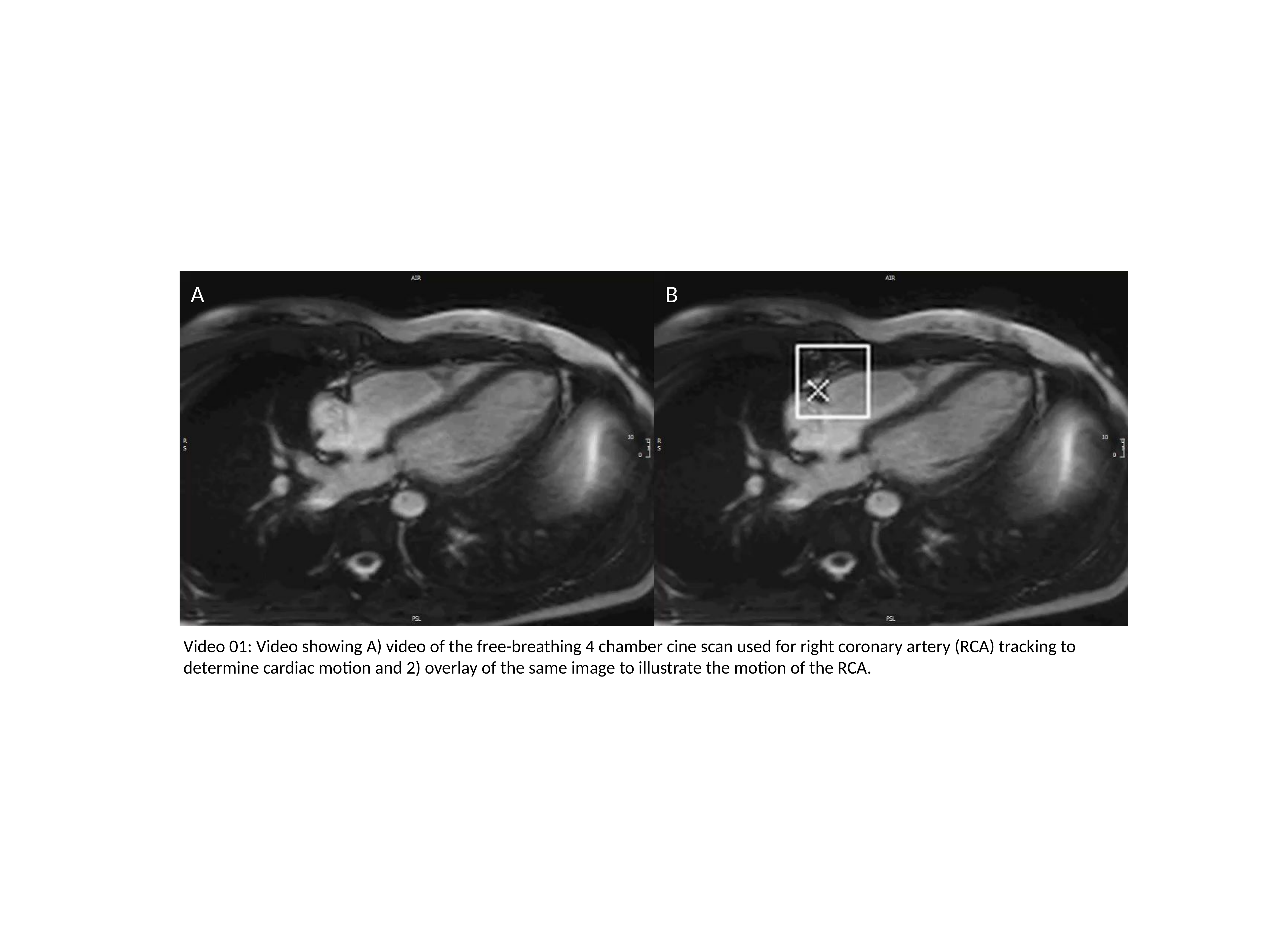

A
B
Video 01: Video showing A) video of the free-breathing 4 chamber cine scan used for right coronary artery (RCA) tracking to determine cardiac motion and 2) overlay of the same image to illustrate the motion of the RCA.
